# Sexually dimorphic leanness and hypermobility in p16^*Ink4a*^/CDKN2A-deficient mice coincides with phenotypic changes in the cerebellum

**DOI:** 10.1038/s41598-019-47676-6

**Published:** 2019-08-01

**Authors:** Kwang H. Kim, Yejin Cho, Jaehoon Lee, Haengdueng Jeong, Yura Lee, Soo In Kim, Chang-Hoon Kim, Han-Woong Lee, Ki Taek Nam

**Affiliations:** 1 Severance Biomedical Science Institute, Brain Korea 21 PLUS Project for Medical Science, Yonsei University College of Medicine, Seoul, 03722 Republic of Korea; 2 Department of Biochemistry, College of Life Science and Biotechnology and Yonsei Laboratory Animal Research Center, Yonsei University, Seoul, 03722 Republic of Korea; 3 Department of Otorhinolaryngology, Korea Mouse Sensory Phenotyping Center, Yonsei University College of Medicine, Seoul, 03722 Republic of Korea

**Keywords:** Cellular neuroscience, Senescence

## Abstract

p16^*Ink4a*^/CDKN2A is a tumor suppressor that critically regulates the cell cycle. Indeed, p16^*Ink4a*^ deficiency promotes tumor formation in various tissues. We now report that p16^*Ink4a*^ deficiency in female mice, but not male mice, induces leanness especially in old age, as indicated by lower body weight and smaller white adipose tissue, although other major organs are unaffected. Unexpectedly, the integrity, number, and sizes of adipocytes in white adipose tissue were unaffected, as was macrophage infiltration. Hence, hypermobility appeared to be accountable for the phenotype, since food consumption was not altered. Histological analysis of the cerebellum and deep cerebellar nuclei, a vital sensorimotor control center, revealed increased proliferation of neuronal cells and improved cerebellum integrity. Expression of estrogen receptor β (ERβ) and PCNA also increased in deep cerebellar nuclei, implying crosstalk between p16^*Ink4a*^ and ERβ. Furthermore, p16^*Ink4a*^ deficiency expands LC3B^+^ cells and GFAP^+^ astrocytes in response to estrogen. Collectively, the data suggest that loss of *p16*^*INK4a*^ induces sexually dimorphic leanness in female mice, which appears to be due to protection against cerebellar senescence by promoting neuronal proliferation and homeostasis via ERβ.

## Introduction

Appropriate control of stem/progenitor cell proliferation is essential to maintain tissue homeostasis in multicellular organisms^1^ and prevent tumor formation. On the other hand, loss of stem/progenitor cell proliferation is implicated in aging and senescence. Although recent medical advances have remarkably extended human life span, there are still challenges to address, including age-associated degenerative diseases like atherosclerosis^2^, diabetes^3^, macular degeneration^4^, osteoporosis^5^, and neurodegenerative diseases such as Parkinson’s disease^6^ and Alzheimer’s disease^7^, both of which are barely reversible.

p16^*Ink4a*^/CDKN2A regulates the cell cycle by slowing the progression from G1 to S phase^8^. For example, p16^*Ink4a*^ binds cyclin-dependent kinase 4/6^9^ to induce retinoblastoma-dependent cell-cycle arrest and prevent tumor progression. p16^*Ink4a*^ is expressed at low levels in normal cells, but is induced by tumorigenic stimuli, and is progressively accumulated with age^10,11^, correlating with reduced proliferative capacity in various tissues^12–14^. These findings imply that cellular senescence due to p16^*Ink4a*^ is closely linked to decreased proliferation of stem/progenitor cells. On the other hand, ablation of p16^*Ink4a*^ induces spontaneous tumorigenesis in mice and increases sensitivity to carcinogens^15,16^. Indeed, p16^*Ink4a*^ is one of the most frequently inactivated genes in human cancers along with p53^17,18^. Hence, p16^*Ink4a*^ appears to be a key regulator of tissue homeostasis.

Sexual dimorphism is the systemic difference in phenotype between males and females^19^. Accordingly, the effects of modifying a sexually dimorphic gene may appear predominantly in only one sex. For example, estrogen, a steroid hormone and the major female sex hormone, is essential for the development and regulation of the female reproduction system. However, estrogen also affects numerous cell processes^20,21^, including neuronal development, as has been documented for years^22^. Many of these effects are mediated by estrogen receptor α (ERα) and estrogen receptor β (ERβ), which are also regulators of neuronal processes such as Purkinje cell differentiation and expression of neurotrophin brain-derived neurotrophic factor^23,24^. Indeed, ERβ knockout mice exhibit impaired spatial learning, neuronal migration, and increased apoptosis^25^. Importantly, estrogen therapy initiated at onset of menopause reduces the risk or delays the onset of Alzheimer’s disease in women^26,27^, suggesting a protective role against neurodegeneration.

We now report that p16^*Ink4a*^*-*deficient female mice gain less weight and accumulate smaller white adipose tissue, effects that became more pronounced with age. These effects are absent from male mice, indicating sexual dimorphism in p16^*Ink4a*^ activity, probably via crosstalk with estrogen receptors. We also investigated the underlying mechanisms, with a view to characterize this previously unreported function of p16^*Ink4a*^.

## Results

### p16^*Ink4a*^ deficiency suppresses weight gain in female mice

**Figure 1:**
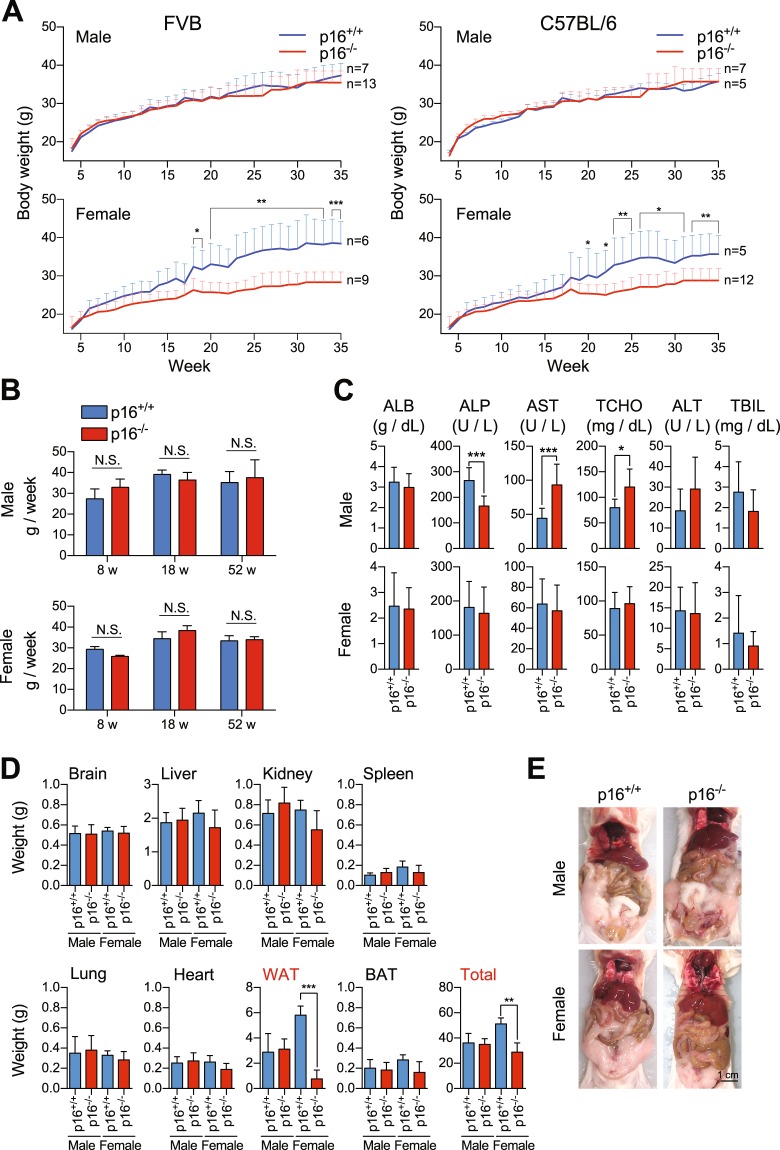
p16^*Ink4a*^ deficiency inhibits weight gain in female mice of different genetic backgrounds. (**A**,**B**) p16^+/+^ and p16^−/−^ male and female mice were weighed weekly at 4 to 35 weeks while housed specific pathogen-free on diets of 25% protein, 13% fat, and 62% carbohydrates (**A)**. Food intake per mouse was measured at 8, 18, and 52 weeks (**B)** (n = 5). (**C)** Blood chemistry analysis of sera from 1 year old p16^+/+^ and p16^−/−^ male and female mice (n = 9). (**D**,**E**) p16^+/+^ and p16^−/−^ male and female mice were sacrificed at 1 year, and the brain, liver, kidney, spleen, lung, heart, white adipose tissue, brown adipose tissue, and total body were weighed **D** (n = 5–9). Mice were also imaged after opening the abdominal cavity (**E**,**A**,**B**,**D)** data are representative of three independent experiments. Results are mean ± SD.

By measuring the weight of p16^*Ink4a*−/−^ (p16^−/−^) FVB mice and littermates (p16^+/+^) at 4–35 weeks from birth, we found that ablation of p16^*Ink4a*^ suppresses weight gain in female but not male mice. This effect became more pronounced with age (Fig. 1A, left panel), and was also observed in C57BL/6 mice (Fig. 1A, right panel). In addition, spontaneous proliferating lesions were more frequent in 1 year-old p16^−/−^ mice (Supplemental Table S1), as verified by a blinded pathologist, and in agreement with previous reports demonstrating that p16^*Ink4a*^ deletion deregulates the cell cycle and promotes tumorigenesis^28^. However, food intake was comparable between p16^+/+^ and p16^−/−^ FVB mice at 8 weeks (before weight differences were observed), 18 weeks (at the time weight differences were observed), and 52 weeks (after body weights started to differ) (Fig. 1B), indicating that p16^*Ink4a*^ deficiency does not induce anorexia due to tumor growth. Blood chemistry data show that p16^*Ink4a*^ deficiency induces some metabolic changes in 1 year old male but not female mice (Fig. 1C). In addition, most organs, including the brain, liver, kidney, spleen, lung, heart, and brown adipose tissue, were comparable in weight in 1 year-old p16^+/+^ and p16^−/−^ FVB male and female mice, but white adipose tissues were dramatically lighter in female but not male p16^−/−^ mice (Fig. 1D,E).

### p16^*Ink4a*^ depletion does not affect adipose tissue physiology

**Figure 2:**
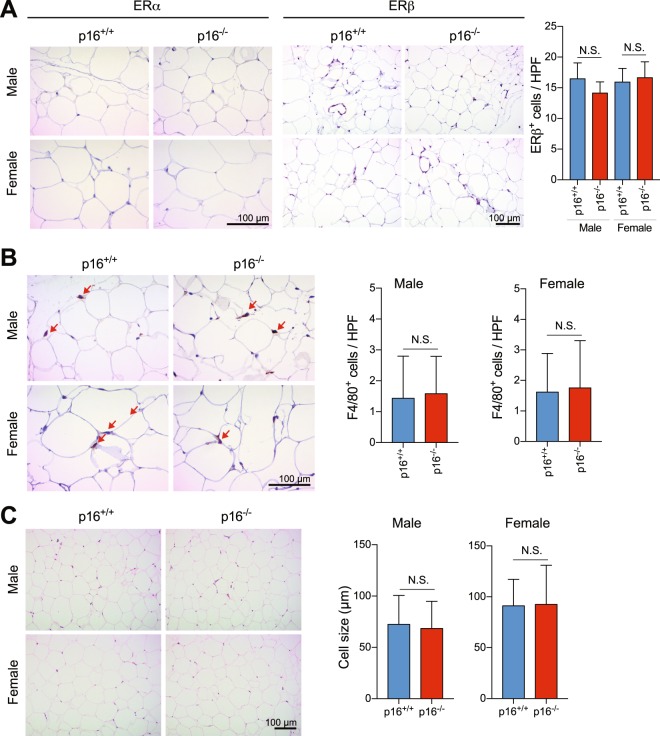
p16^*Ink4a*^ deficiency does not alter homeostasis in white adipose tissue. (**A**) White adipose tissues from p16^+/+^ and p16^−/−^ FVB male and female mice were stained with anti-ERα (left panel) and anti-ERβ (right panel). ERβ^+^ cells (brown) were quantified at high power (n = 15). (**B**) Number of F4/80^+^ cells (brown) (n = 15). (**C**) Adipocyte size (n = 15). HPF, high power field. Results are mean ± SD.

Since estrogen is a major regulator of adipose tissue in female animals^29^, we examined whether p16^*Ink4a*^ depletion may affect the expression of estrogen receptors or the number of cells expressing estrogen receptors. Thus, paraffinized sections of white adipose tissue from 1 year-old p16^+/+^ and p16^−/−^ FVB male and female mice were immunohistochemically stained with antibodies to ERα and ERβ. While ERα-expressing cells were not detected (Fig. 2A, left), ERβ-expressing cells were found throughout the entire tissue section. However, the number of ERβ^+^ cells was comparable between p16^+/+^ and p16^−/−^ mice of both sexes (Fig. 2A, right). F4/80^+^ macrophages, which infiltrate adipose tissues in obese organisms and are significantly and positively correlated with adipocyte size^30^, were also comparable in number between p16^+/+^ and p16^−/−^ mice (Fig. 2B). In addition, adipocytes are similar in size among all animals (Fig. 2C), indicating that p16^*Ink4a*^ deficiency does not affect adipose tissues *per se*.

### p16^*Ink4a*^ deficiency promotes neural cell proliferation in female mice

**Figure 3:**
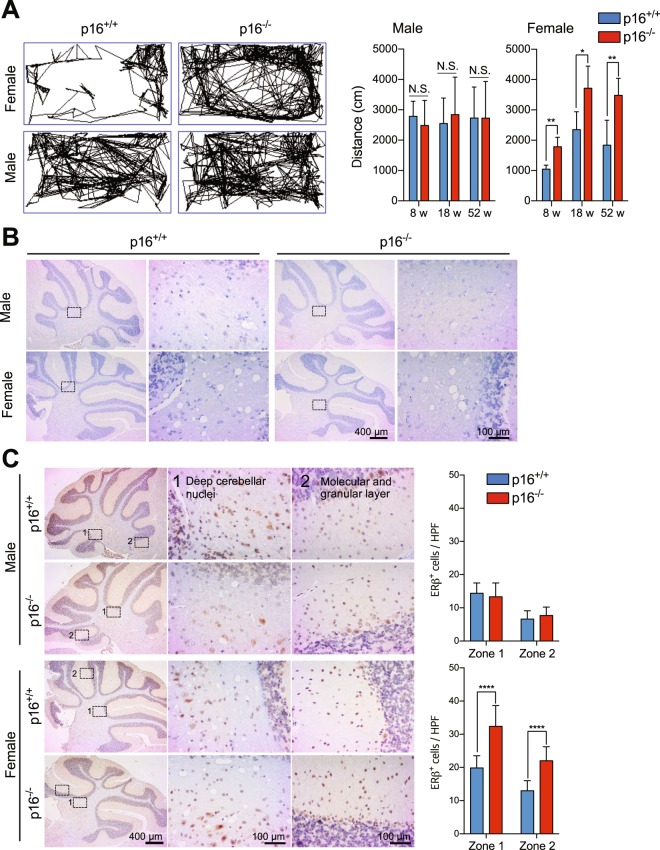
Mouse mobility and distribution of estrogen receptors in the cerebellum. (**A**) Movements of 1 year-old p16^+/+^ and p16^−/−^ FVB mice were tracked by video for 3 min in a test cage (left images) and total distances were calculated (right graph) (n = 8). (**B**,**C**) Cerebellum sections from p16^+/+^ and p16^−/−^ FVB male and female mice were stained with anti-ERα (**B)** or anti-ERβ (**C)** and ERβ^+^ cells (brown) were quantified (n = 15). HPF, high power field. Results are mean ± SD.

We observed that female p16^−/−^ mice are hyperactive in comparison to female p16^+/+^ mice. Indeed, movement tracking for 3 min revealed that the former moved across 2-fold longer distance than the latter, suggesting that p16^*Ink4a*^ deficiency may be linked to hypermobility in females. In contrast, mobility was comparable in male p16^+/+^ and p16^−/−^ mice (Fig. 3A and Supplemental Fig. 1).

**Figure 4:**
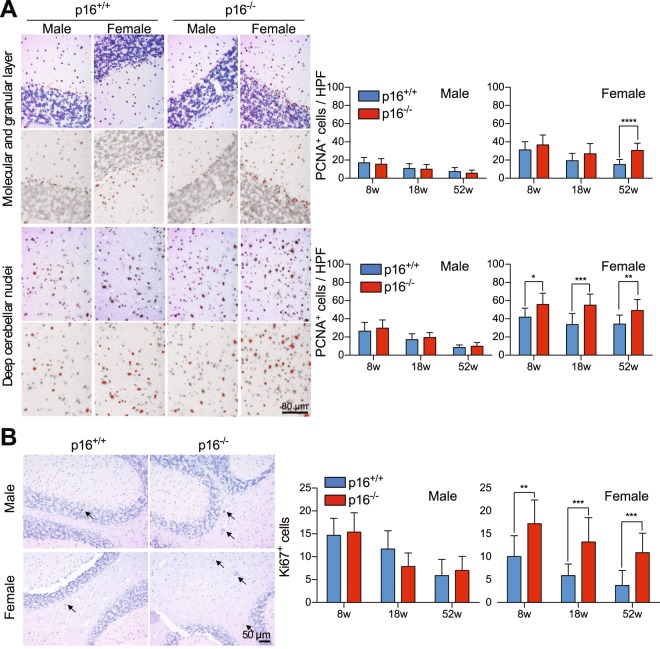
p16^*Ink4a*^ deficiency promotes proliferation of neural cells in the cerebellum of female mice only. (**A**,**B**) Cerebellum sections from p16^+/+^ and p16^−/−^ FVB male and female mice were stained with anti-PCNA (**A**, n = 15) and anti-Ki67 (**B**, n = 9–15), and stained cells in the cerebellum and in deep cerebellar nuclei (brown) were counted in Image J as red dots in a gray background. HPF, high power field. Results are mean ± SD.

**Figure 5:**
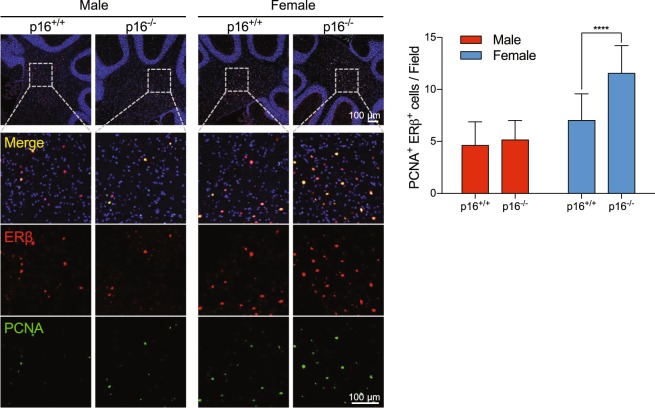
Estrogen signaling promotes the proliferation of neural cells in deep cerebellar nuclei region of female p16^*Ink4a*^ knockout mice. Sections of deep cerebellar nuclei from p16^+/+^ and p16^−/−^ FVB male and female mice were stained with anti-ERβ (red) and anti-PCNA (green) to quantify ERβ^+^ PCNA^+^ cells/field. Nuclei were stained with DAPI (blue) (n = 15–21). Results are mean ± SD.

ERα-expressing cells were observed by immunohistochemistry in the thalamus (Supplemental Fig. 2) but not in the cerebellum, which coordinates motor activities^31^ (Fig. 3B). In contrast, ERβ-expressing cells were pervasive throughout the cerebellum, including in the molecular and granular layer and in deep cerebellar nuclei (Fig. 3C). ERβ^+^ cells were more abundant in female (~27 cells/high-power field in the molecular and granular layer, ~18 cells/high-power field in deep cerebellar nuclei) than in male mice (~18 cells/high-power field in the molecular and granular layer, ~12 cells/high-power field in deep cerebellar nuclei), but comparable between p16^+/+^ and p16^−/−^ male mice (Fig. 3C). However, ERβ^+^ cells were more abundant in female p16^−/−^ mice. In addition, cells expressing proliferating cell nuclear antigen (PCNA), a marker of proliferation, were significantly more abundant in the same tissues in female p16^−/−^ mice than in female p16^+/+^ mice at 18 and 52 weeks (Fig. 4A), even though comparable in number among all male mice. Similarly, cells expressing Ki67, also a marker of proliferation, accumulated at all time points examined in female p16^−/−^ mice only (Fig. 4B). Finally, double immunofluorescence staining indicated that ERβ^+^ PCNA^+^ cells in deep cerebellar nuclei were significantly more abundant in p16^−/−^ female mice than in wild type female mice, but comparable among all male mice (Fig. 5), suggesting that suppression of p16^*Ink4a*^ enhances mobility in female mice, possibly via estrogen-associated neural cell proliferation.

### p16^*Ink4a*^ deficiency protects female cerebella from aging-associated deterioration

**Figure 6:**
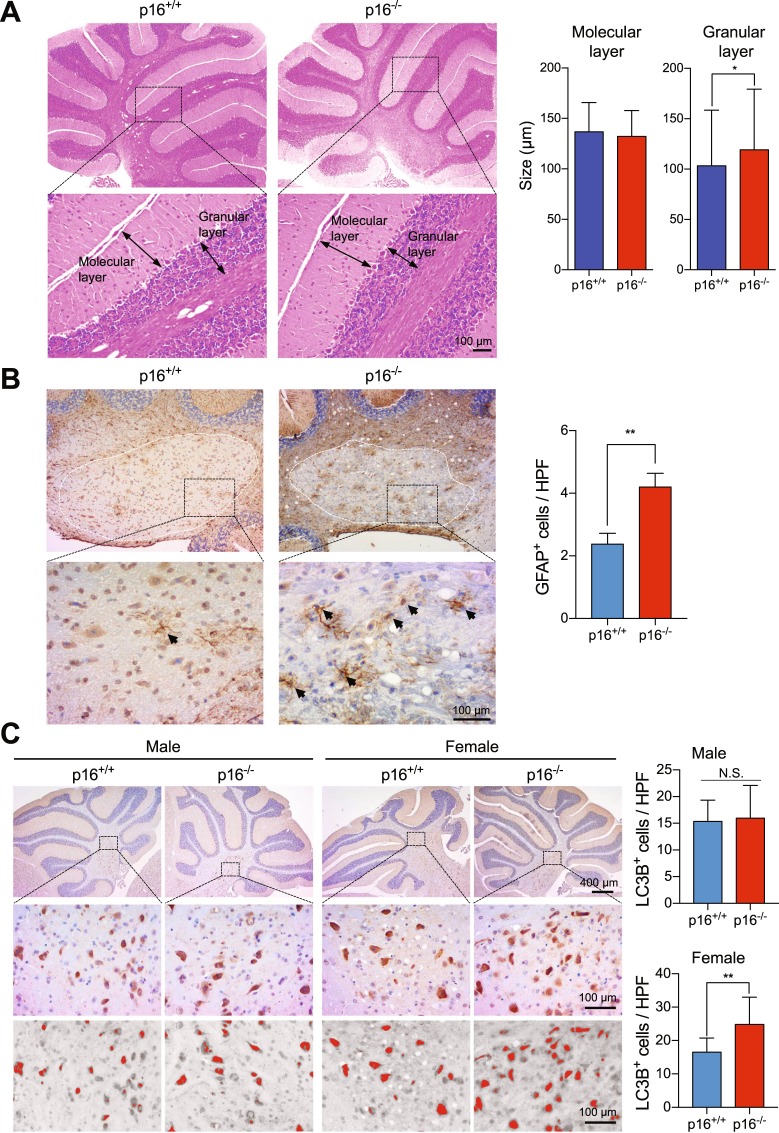
p16^*Ink4a*^ regulates senescence in the cerebellum. (**A**) Size of the granular (GL) and molecular layer (ML) in the cerebellum of p16^+/+^ and p16^−/−^ female FVB mice (n = 15). (**B**) Sections of deep cerebellar nuclei from p16^+/+^ and p16^−/−^ female FVB mice were stained with anti-GFAP, and GFAP^+^ cells (brown, black arrow head) were quantified at high power (right panel) (n = 9). (**C**) Cerebellum sections from p16^+/+^ and p16^−/−^ FVB male and female mice were stained with anti-LC3B, and LC3B^+^ cells (brown) were counted in ImageJ as red dots in a gray background (n = 15). HPF, high power field. Results are mean ± SD.

We investigated whether p16^*Ink4a*^ deficiency may protect the cerebellum from deterioration, which is typically associated with age or neurodegeneration^32^. Loss of p16^*Ink4a*^ slightly expanded the granular layer, although the size of the entire cerebellum was not affected (Fig. 6A). In addition, astrocytes, the major cell type that maintains brain integrity^33^, were more abundant in the deep cerebellar nuclei region of p16^−/−^ mice, as assessed by staining for GFAP (Fig. 6B, white dotted line). Nevertheless, p16^*Ink4a*^ deficiency did not enhance astrocyte proliferation as assessed by staining for PCNA (Supplemental Fig. 3).

Autophagy, which is essential for survival of neural cells and is impaired in neurodegenerative disorders and during senescence^34,35^, is more robust in p16^−/−^ female mice than in female p16^+/+^ mice, as suggested by the accumulation of LC3B^+^ cells in the deep cerebellar nuclei region (~19 cells/high-power field in p16^+/+^, ~27 cells/high-power field in p16^−/−^). However, autophagy was comparable among male mice (Fig. 6C). These results suggest that p16^*Ink4a*^ deficiency may protect the cerebellum by activating autophagy.

## Discussion

We found that deletion of p16^*Ink4a*^, a cell cycle regulator, attenuates age-associated obesity in female mice by inducing hypermobility. Protection against deterioration of the cerebellum, a motor center, appears to be essential to this phenotype, since ablation of p16^*Ink4a*^ promotes neuronal cell proliferation and increases autophagic cells and astrocytes in this region of the brain. However, p16^*Ink4a*^ deficiency was not similarly beneficial in male mice, presumably as a result of sexual dimorphism, as seen in many different phenotypes^19,36^. The dimorphism is likely mediated by estrogen, which is essential to female development but which also regulates brain development and homeostasis, especially in females. Estrogen receptors are also more abundantly expressed in the female brain than in the male brain^37^, in line with our results.

In specific pathogen-free breeding, mice consume feed continuously in confined spaces with little mobility, such that the abdominal cavity becomes full of fat in most of these mice after a year or so. Remarkably, this phenomenon was not observed in p16^*Ink4a*^ -deficient female mice. Hence, we hypothesized that ablation of p16^*Ink4a*^ may affect the development of white adipose tissue or induce metabolic changes. However, we did not find differences in the number of adipocytes expressing estrogen receptors, the size of adipocytes, and the number of infiltrating F4/80^+^ macrophages, which is significantly and positively correlated with both adipocyte size and body weight^30^. Therefore, ablation of p16^*Ink4a*^ has little effect on adipocyte metabolism. On the other hand, senescence of the cerebellum reduces motility, which can also lead to obesity^38,39^. Remarkably, we found that suppression of p16^*Ink4a*^ increases proliferation of neuronal cells, delays senescence in the cerebellum, and enhances mobility. In addition, p16^−/−^ female mice were also more responsive to peripheral stimulation than wild type (data not shown).

Homeostasis is critical in the brain, an organ with less proliferation but longer cell life, than in other tissues. GFAP^+^ astrocytes, which regulate neurotransmitter trafficking and recycling, ion homeostasis, energy metabolism, and defense against oxidative stress to maintain brain homeostasis^33^, were significantly more abundant in the former (Fig. 6B). Also, autophagy is an important process where age-related loss of autophagy accelerates the progression of neurodegenerative disease due to accumulation of toxic protein aggregates in neurons^40^, and LC3 was also highly expressed on astrocytes^41^. Furthermore, neural stem cells (NSCs) are required for generating neural cell, and autophagy plays an important role in stemness and neurogenesis of NSCs^42,43^. Notably, LC3^+^ neurons were more abundant in the deep cerebellar nuclei region of p16^−/−^ female mice than in wild type female mice (Fig. 6C). We also found that ERβ^+^ cells and proliferating ERβ^+^ cells were increased in p16^−/−^ female (Figs 3 and 5). ERβ is important for maintaining neural homeostasis; in fact, ERβ deficiency in adult mouse has shown increased vulnerability to neurodegeneration^44^. In addition, ERβ signaling is important for differentiation of neuronal precursors^45^.

Therefore, there are two possibilities for the increased LC3^+^ cells in p16^−/−^ condition: (1) promoted proliferation of ERβ^+^ NSCs, which were highly expressed in LC3, and (2) increased ERβ^+^ neural progenitor cells were differentiated into neuronal cells, such as LC3^+^ astrocyte, in response to estrogen signal following the loss of p16^*Ink4a*^. We need to further identify the role of the stem, progenitor, or differentiated neuron cells controlled by p16^*Ink4a*^ in response to estrogen.

As previously reported, loss of p16^*Ink4a*^ clearly induces development of sporadic cancers. However, tumors develop only after a considerable amount of time even if p16^*Ink4a*^ is mutated, so modulating p16^*Ink4a*^ at an advanced age to prevent dementia may still prove beneficial on balance. Alternatively, neuron-specific regulation of p16^*Ink4a*^ and enhanced estrogen signaling may help overcome various neurological diseases.

## Materials and Methods

### Animal models

Animal experiments were approved by the Institutional Animal Care and Use Committee of Yonsei University (IACUC number: 2015–0116) and were compliant with the Guide for the Care and Use of Laboratory Animals. FVB and C57BL/6 mice were purchased from The Jackson Laboratory, maintained in a specific pathogen-free barrier facility under a 12 h light cycle, and provided PicoLab^®^ Rodent Diet 20 (LabDiet, St. Louis, MO USA).

TALENs were designed to cleave downstream of the p16^*Ink4a*^ start codon to introduce frameshift mutations and thus knock out the gene. Plasmids encoding left- and right-TALENs that recognize the p16^*Ink4a*^ sequences 5′-TGCATGACGTGCGGGCACTG-3′ and 5′-GTTTCGCCCAACGCCCCGAA-3′, respectively, were obtained from ToolGen. TALEN mRNAs were synthesized using mMESSAGE mMACHINE T7 Ultra Kit (Thermo Fisher Scientific, Waltham, MA USA) following the manufacturer’s instructions, and diluted in buffer containing 0.25 mM EDTA and 10 mM Tris pH 7.4 and pretreated with diethyl pyrocarbonate. These mRNAs were injected at 50 ng/μL into the cytoplasm of one-cell embryos using a piezo-driven manipulator (Prime Tech, Tsuchiura, Japan), as previously described^46^. Subsequently, embryos were transferred into foster mothers to generate F0 mice, which were screened by PAGE-PCR as previously described^47^, using genomic DNA obtained from newborns. PCR products of candidate knockout mice were then cloned in T-Blunt PCR Cloning Vector (SolGent, Deajeon, South Korea), and mutations were validated by direct sequencing (Cosmogenetech, Seoul, South Korea). Mice with the earliest premature stop codons were selected as p16^*Ink4a*^ knockout strain.

p16^+/+^ and p16^−/−^ FVB or C57BL/6 mice were maintained by crossing with p16^+/−^ × p16^+/−^. Four weeks after birth, offspring were separated from mother cage and genotyped with the following primers: 5′-GAGGAGAGCCATCTGGAG-3′ and 5′-CCTTGCCTACCTGAATCG-3′. p16^+/+^ was amplified to 153 base pair and p16^−/−^ was amplified to 133 bp after PCR reaction.

### Antibodies

Antibodies to F4/80 (Cat no: ab6640), Ki67 (Cat no: ab16667), LC3B (Cat no: ab48394), GFAP (Cat no: ab7260) were obtained from Abcam. Antibodies to ERα (Cat no: sc-390244) and PCNA (Cat no: sc-56) were obtained from Santa Cruz Biotechnology, while anti-ERβ (Cat no: PA1-310B) was obtained from Thermo Fisher Scientific.

### Assessment of mouse mobility

Mouse mobility was assessed according to Cho *et al*.^48^. Briefly, male and female p16^+/+^ and p16^−/−^ FVB mice were placed in standard polypropylene rodent breeding cages with standard-depth wood chip bedding, and which were not covered during experiments to prevent internal saturation with odorants. A video camera (HD lens 720P 30FPS Auto Widescreen) was fixed vertically about 1 m above the cage and linked to a Smart 3.0 video-tracking system (Harvard apparatus^©^, Holliston, MA USA). Mouse behavior was recorded and automatically tracked for 3 min.

### Blood chemistry

One-year old p16^+/+^ and p16^−/−^ male and female FVB mice were sacrificed using by CO_2_ gas, and total blood was immediately obtained from the heart using 26G 1 ml syringe. To isolate serum form of total blood, blood samples were placed in the MiniCollect^®^ tube (Greiner Bio-One, Kremsmünster, Austria) and centrifugation 1 × 10^4^ rpm for 5 min at room temperature. Sera (10 μL) from 1 year-old p16^+/+^ and p16^−/−^ male and female FVB mice were analyzed on a DRI-chem 4000i (Fuji, Minato, Japan) automated clinical chemistry analyzer to quantify changes in albumin (ALB), alkaline phosphatase (ALP), aspartate aminotransferase (AST), total cholesterol (TCHO), alanine aminotransferase (ALT), and total bilirubin (TBIL).

### Food intake

Each of the two mice groups were bred in a single cage, and were supplied with 200 g of PicoLab^®^ Rodent Diet 20. We measured the amount of food left on a digital scale, and the amount of reduced food was defined as the amount of food the mice ingested. The measurement was performed every other day for two weeks, and the results were converted to the amount of food consumed per week. The amount of food powder that fell to the bottom of the cage was negligible in this measurement, since its amount had no significant effect on the result.

### Immunohistochemistry and immunofluorescence

For immunohistochemistry, tissues were fixed with ice-cold 4% paraformaldehyde in phosphate-buffered saline and mounted in paraffin blocks by conventional methods. Samples were sectioned at 3 μm, deparaffinized with xylene three times for every 20 min, 100% EtOH three times for every 10 min, 90% EtOH two times for every 10 min, and 75% EtOH for 10 min, and then rehydrated in PBS. Antigens were then retrieved for 15 min at high pressure in Target Retrieval Solution (Dako, Santa Clara, CA USA). Subsequently, specimens were chilled on ice for 1 h, washed with PBS three times for 5 min, and blocked with 3% H_2_O_2_ in PBS for 30 min to quench endogenous peroxidase. Slides were washed again with PBS, blocked for 2 h at room temperature with Serum-Free Protein Block (Dako, Santa Clara, CA USA), probed at 4 °C overnight with primary antibodies (1/1 000 dilution), stained for 30 min with anti-mouse (Dako, Santa Clara, CA USA) or anti-rabbit IgG (Dako, Santa Clara, CA USA) conjugated to horseradish peroxidase, and developed with Liquid DAB + Substrate Chromogen System (Dako, Santa Clara, CA USA). Finally, specimens were counterstained with Mayer’s hematoxylin (Dako, Santa Clara, CA USA) and mounted with Shandon Synthetic Mount (Thermo Fisher Scientific, Waltham, MA USA).

For immunofluorescence, tissues samples were blocked as described, probed at 4 °C overnight with primary antibodies (1/1 000 dilution), labeled for 2 h with anti-mouse IgG conjugated to Alexa 488 (Thermo Fisher Scientific, Waltham, MA USA) or with anti-rabbit IgG conjugated to cyanine5 (Thermo Fisher Scientific, Waltham, MA USA), and stained with DAPI for 15 min. Finally, slides were mounted with ProLong™ Gold antifade reagent (Thermo Fisher Scientific, Waltham, MA USA), imaged on an LSM 700 confocal microscope (Zeiss, Oberkochen, Germany), and analyzed using Zeiss Zen Blue Edition.

### Statistical analysis

Statistical significance between the two groups was determined by unpaired nonparametric Mann-Whitney test. P value < 0.05 was considered statistically significant, and significance was designated with asterisks (N.S., not significant; *P < 0.05; **P < 0.01; ***P < 0.001; ****P < 0.0001). All data were evaluated in GraphPad Prism version 7.0d (GraphPad, San Diego, CA USA) for statistical analyses.

## Supplementary information

supplementary information
