# Supplementary material for: Sexually dimorphic leanness and hypermobility in p16Ink4a/CDKN2A-deficient mice coincides with phenotypic changes in the cerebellum
Source: Sci Rep. 2019 Aug 1;9:11167. doi: 10.1038/s41598-019-47676-6 (PMC6671985; doi:10.1038/s41598-019-47676-6)
Supplement: Supplementary file 1 — supplementary information [file 41598_2019_47676_MOESM1_ESM.pdf]

Sexually dimorphic leanness and hypermobility in p16^{Ink4a}/CDKN2A-deficient mice coincides with phenotypic changes in the cerebellum

Kwang H. Kim[1] • Yejin Cho[1] • Jaehoon Lee[2] • Haengdueng Jeong[1] • Yura Lee[1] • Soo In Kim[3] • Chang-Hoon Kim[3] • Han-Woong Lee[2] • Ki Taek Nam[1]

[1] Severance Biomedical Science Institute, Brain Korea 21 PLUS Project for Medical Science, Yonsei University College of Medicine, Seoul 03722, Republic of Korea

[2] Department of Biochemistry, College of Life Science and Biotechnology and Yonsei Laboratory Animal Research Center, Yonsei University, Seoul 03722, Republic of Korea

[3] Department of Otorhinolaryngology, Korea Mouse Sensory Phenotyping Center, Yonsei University College of Medicine, Seoul 03722, Republic of Korea

Address correspondence to: Ki Taek Nam, DVM, PhD, Severance Biomedical Science Institute, Yonsei University College of Medicine, 50-1 Yonsei-ro, Seodaemun-gu, Seoul 03722, Korea, Tel: 82-2-2228-0754, Fax: 82-2-2227-8129, E-mail: kitaek@yuhs.ac.

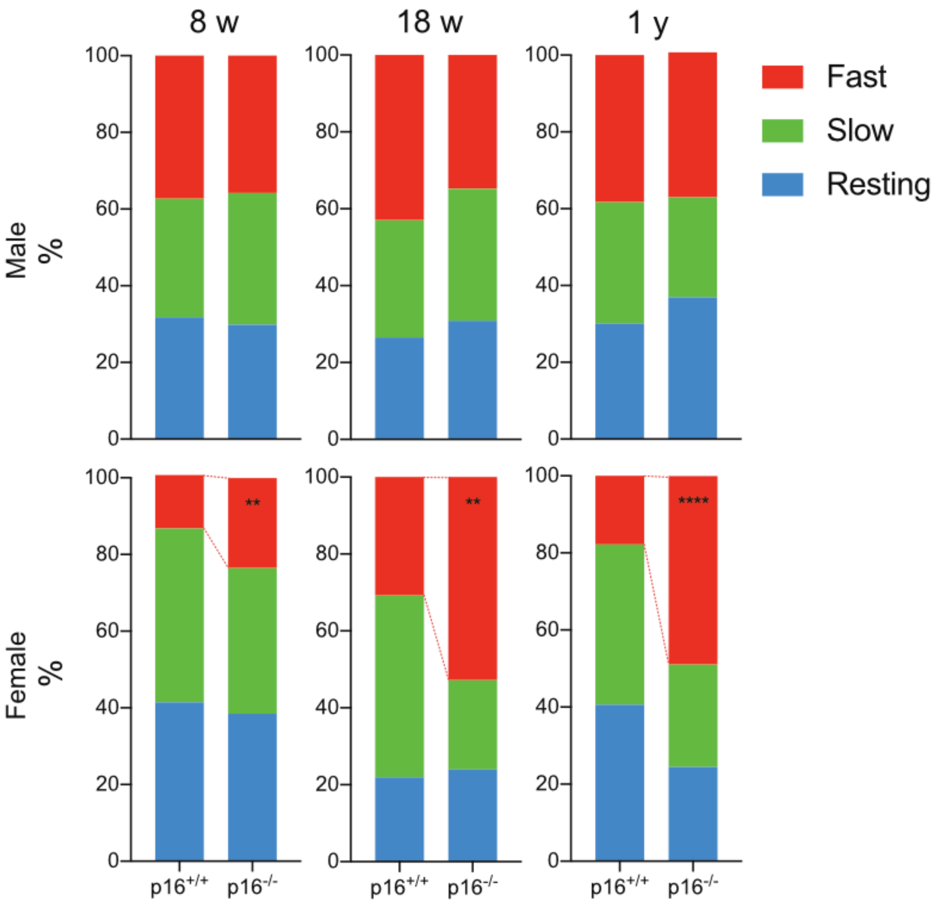

Supplemental Figure 1. Female p16^{Ink4a}-deficient mice are more mobile. Movements of p16$^{+/+}$ and p16$^{-/-}$ FVB male and female mice were tracked for 3 min to determine percentage of fast movement, slow movement, and resting time. Results are mean ± SD.

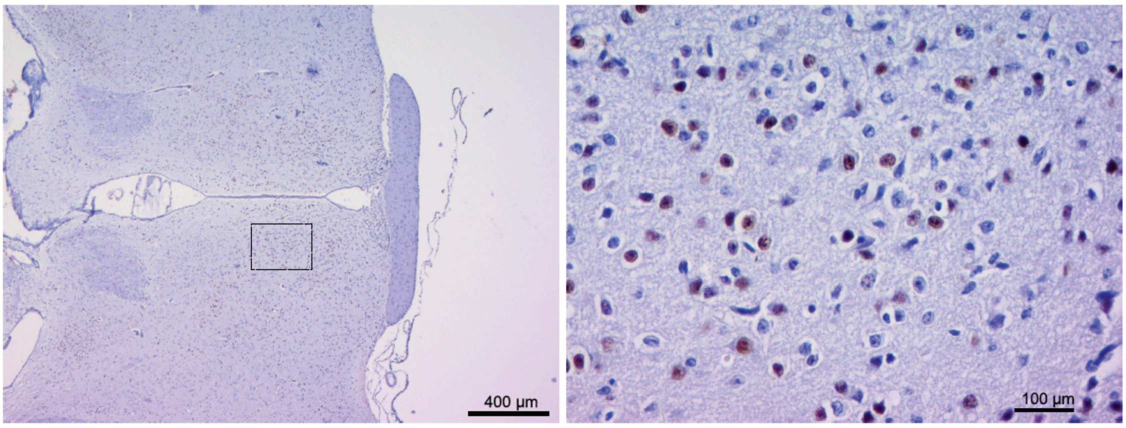

Supplemental Figure 2. ERα distribution in the mouse brain. Whole brain sections from wild type FVB mice were stained with anti-ERα. ERα$^+$ cells (brown) were observed in the thalamus and hypothalamus.

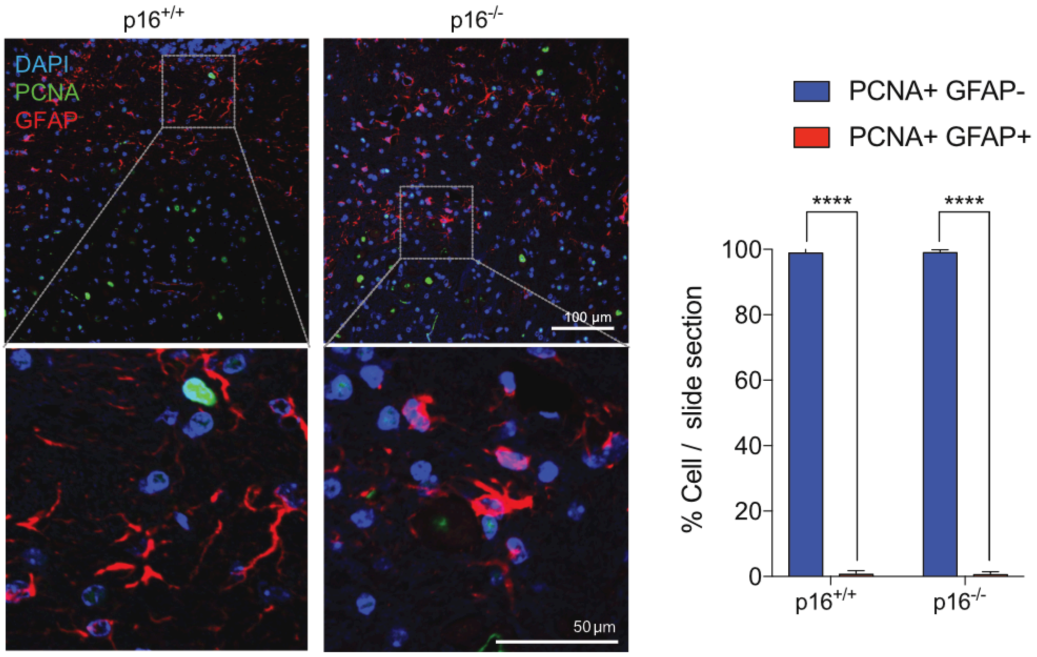

Supplemental Figure 3. Astrocytes are not proliferating. Sections of deep cerebellar nuclei from $p16^{+/+}$ and $p16^{-/-}$ FVB female mice were stained with anti-GFAP (red) and anti-PCNA (green) (n=15). Nuclei were stained with DAPI (blue). Results are mean ± SD. (****, $P < 0.0001$).

Supplemental Table 1. Characteristics of $p16^{-/-}$ mice at 1 year as assessed by a pathologist (n = 54)

Characteristics, Male (n = 29)	Data	
	$p16^{+/+}$ (n = 9)	$p16^{-/-}$ (n = 20)
Proliferating		
Liver, n (%)		
Multifocal leukemia	0 (0.00)	1 (5.00)
Adenoma	0 (0.00)	1 (5.00)
Kidney, n (%)		
Lymphoma	0 (0.00)	1 (5.00)
Lung, n (%)		
Leukemia	3 (33.33)	4 (20.00)
Alveolar hyperplasia	0 (0.00)	11 (55.00)
Bronchoalveolar adenoma	4 (44.44)	4 (20.00)
Lymphoma	0 (0.00)	2 (10.00)
Papillary adenocarcinoma	1 (11.11)	1 (5.00)
Adrenal gland, n (%)		
Subcapsular hyperplasia	1 (11.11)	2 (10.00)
Pheochromocytoma	0 (0.00)	2 (10.00)
Cortical hyperplasia	0 (0.00)	1 (5.00)
Subcapsular adenoma	0 (0.00)	1 (5.00)
Cortical adenoma	2 (22.22)	0 (0.00)
Stomach, n (%)		
Foveolar hyperplasia	3 (33.33)	7 (35.00)
Lymphoma	0 (0.00)	1 (5.00)
Adenoma	1 (11.11)	2 (10.00)
Adenocarcinoma	0 (0.00)	6 (30.00)
Urinary bladder, n (%)		
Diffuse transitional cell hyperplasia	0 (0.00)	1 (5)
Testis, n (%)		
Hemangiosarcoma	0 (0.00)	2 (10.00)
Eye, n (%)		
Harderian gland adenocarcinoma	0 (0.00)	1 (5.00)
Non-proliferating		
Liver, n (%)		
Multifocal altered cell foci	3 (33.33)	11 (55.00)
Lymphocytic focal inflammation	0 (0.00)	1 (5.00)
Focal inflammation	0 (0.00)	2 (10.00)
Focal necrosis	1 (11.11)	1 (5.00)
Multifocal necrosis	1 (11.11)	4 (20.00)
Kidney, n (%)		
CPN	4 (44.44)	10 (50.00)
Inflammation	0 (0.00)	1 (5.00)
Lung, n (%)		
Focal inflammation	0 (0.00)	2 (10.00)
Stomach, n (%)		
Cystic dilatation	0 (0.00)	4 (20.00)
Dysplasia	1 (11.11)	0 (0.00)
Testis, n (%)		
Testicular atrophy	1 (11.11)	1 (5.00)
Prostatitis	0 (0.00)	1 (5.00)

Characteristics, Female (n = 25)	Data	
	$p16^{+/+}$(n = 6)	$p16^{-/-}$ (n = 19)
Proliferating		
Liver, n (%)		
Multifocal leukemia	0 (0.00)	1 (5.26)
Kidney, n (%)		
Leukemia	0 (0.00)	2 (10.52)
Lung, n (%)		
Leukemia	4 (66.67)	2 (10.52)
Alveolar hyperplasia	2 (33.33)	14 (73.68)
Bronchoalveolar carcinoma	1 (16.67)	2 (10.52)
Adrenal gland, n (%)		
Subcapsular hyperplasia	0 (0.00)	1 (5.26)
Stomach, n (%)		
Foveolar hyperplasia	4 (66.67)	6 (31.57)
Colon, n (%)		
Rectal adenoma	0 (0.00)	1 (5.26)
Uterus, n (%)		
Cystic hyperplasia	5 (83.33)	14 (73.68)
Leivy sarcoma	0 (0.00)	1 (5.26)
Ear, n (%)		
Fibrosarcoma	0 (0.00)	1 (5.26)
Non-proliferating		
Liver, n (%)		
Multifocal altered cell foci	0 (0.00)	4 (21.05)
Lymphocytic focal inflammation	2 (33.33)	1 (5.26)
Focal inflammation	0 (0.00)	4 (21.05)
Multifocal inflammation	0 (0.00)	3 (15.80)
Focal necrosis	1 (16.67)	1 (5.26)
Kidney, n (%)		
CPN	3 (50.00)	5 (26.31)
Inflammation	0 (0.00)	1 (5.26)
Hydronephrosis	0 (0.00)	1 (5.26)
Focal lymphocyte inflammation	0 (0.00)	1 (5.26)
Lung, n (%)		
Focal inflammation	1 (16.67)	1 (5.26)
Hemosiderosis	0 (0.00)	1 (5.26)
Cystic dilatation	0 (0.00)	1 (5.26)
Multifocal inflammation	0 (0.00)	1 (5.26)
Adrenal gland, n (%)		
Lipidosis in X-zone	0 (0.00)	1 (5.26)
Stomach, n (%)		
Cystic dilatation	1 (16.67)	1 (5.26)
Dysplasia	1 (16.67)	1 (5.26)
Colon, n (%)		
Altered cell foci	0 (0.00)	1 (5.26)
